# Targeting Aberrantly Elevated Sialyl Lewis A as a Potential Therapy for Impaired Endometrial Selection Ability in Unexplained Recurrent Miscarriage

**DOI:** 10.3389/fimmu.2022.919193

**Published:** 2022-06-28

**Authors:** Zhi Ma, Huixia Yang, Mirjana Kessler, Markus Sperandio, Sven Mahner, Udo Jeschke, Viktoria von Schönfeldt

**Affiliations:** ^1^ Department of Obstetrics and Gynaecology, University Hospital, Ludwig-Maximilians-Universität Munich, Munich, Germany; ^2^ Biomedical Center (BMC), Institute for Cardiovascular Physiology and Pathophysiology, Walter Brendel Center for Experimental Medicine (WBex), Faculty of Medicine, Ludwig-Maximilians-Universität Munich, Munich, Germany; ^3^ Department of Obstetrics and Gynaecology, University Hospital Augsburg, Augsburg, Germany

**Keywords:** Lewis antigens, sLeA, LeX, FUT3, recurrent miscarriage

## Abstract

**Background:**

Carbohydrate Lewis antigens including sialyl Lewis A (sLeA), sialyl Lewis X (sLeX), Lewis X (LeX), and Lewis Y (LeY) are the commonest cell surface glycoconjugates that play pivotal roles in multiple biological processes, including cell adhesion and cell communication events during embryogenesis. SLeX, LeY, and associated glycosyltransferases ST3GAL3 and FUT4 have been reported to be involved in human embryo implantation. While the expression pattern of Lewis antigens in the decidua of unexplained recurrent miscarriage (uRM) patients remains unclear.

**Methods:**

Paraffin-embedded placental tissue slides collected from patients experiencing early miscarriages (6–12 weeks) were analyzed using immunohistochemical (IHC) and immunofluorescent (IF) staining. An *in vitro* assay was developed using endometrial cell line RL95-2 and trophoblast cell line HTR-8/SVneo. Modulatory effect of potential glycosyltransferase on Lewis antigens expression was investigated by target-specific small interfering RNA (siRNA) knockdown in RL95-2 cells. HTR-8/SVneo cells spheroids adhesion assay was applied to investigate the intrinsic role of Lewis antigens in the abnormal implantation process of uRM. The expression of Lewis antigens in RL95-2 cells in response to the treatment with pro-implantation cytokine IL-1β was further measured by flow cytometry and immunocytochemical (ICC) staining.

**Results:**

IHC staining revealed that Lewis antigens are mainly expressed in the luminal and glandular epithelium, IF staining further indicated the cellular localization at the apical membrane of the epithelial cells. FUTs, ST3GALs, and NEU1 located in both stromal and epithelial cells. We have found that the expression of sLeA, LeX, FUT3/4, and ST3GAL3/4 are significantly upregulated in the RM group, while FUT1 is downregulated. SLeX, LeY, ST3GAL6, and NEU1 showed no significant differences between groups. FUT3 knockdown in RL95-2 cells significantly decreased the expression of sLeA and the spheroids adhesion to endometrial monolayer. Anti-sLeA antibody can remarkably suppress both the basal and IL-1β induced adhesion of HTR-8/SVneo spheroids to RL95-2 cells monolayer. While further flow cytometry and ICC detection indicated that the treatment of RL95-2 cells with IL-1β significantly increases the surface expression of LeX, but not sLeA.

**Conclusions:**

SLeA, LeX, and pertinent glycosyltransferase genes FUT1/3/4 and ST3GAL3/4 are notably dysregulated in the decidua of uRM patients. FUT3 accounts for the synthesis of sLeA in RL95-2 cells and affects the endometrial receptivity. Targeting aberrantly elevated sLeA may be a potential therapy for the inappropriate implantation in uRM.

## Introduction

Miscarriage is the most common and frustrating disorder of early pregnancy. An estimated 9-20% of clinically recognized pregnancies end in spontaneous loss, and up to 50% of all conceptions are lost at preclinical stages as biochemical loss or implantation failure (1). Recurrent miscarriage (RM), the failure of two or more pregnancies prior to 20 weeks, affects 1-4% of all couples trying to conceive (1, 2). Known risk factors associated with RM are maternal age, previous pregnancy losses, genetic abnormalities, uterine anomalies, and autoimmune disorders. Even after a thorough evaluation, no causes can be identified in more than 50% of RM couples (3), which are defined as unexplained RM (uRM).

Accumulating studies indicate that uRM is associated with the impaired selection ability of decidual cells, allowing embryos of poor viability to implant inappropriately (4–8). Decidualized endometrial stromal cells (ESCs) were reported to be able to sense an arresting embryo, and response with shutting down the production of pro-implantation modulator IL-1β, preventing the implantation of incompetent embryos (9). While ESCs of uRM patients cannot discriminate between high- and low-quality embryos (10).

Sialyl Lewis A (sLeA) and sialyl Lewis X (sLeX) are commonly found on the surface of many types of cancer cells, facilitating the hematogenous metastasis of these cells through interaction with endothelial cells (11). Studies also reveal that sLeA/X are abundant at the endometrium during implantation stage (12). SLeX is the major terminal carbohydrate sequence on zona pellucida, mediating human sperm-egg binding (13). Specific fucosyltransferases (FUTs), sialyltransferases (STs), and neuraminidases (NEUs) are involved in the synthesis of sLeA/X and related epitopes (11, 14–17). Despite the significant roles of sLeX and LeY in normal blastocyst implantation have been investigated in several *in vitro* and animal models (18–20), their expression pattern together with other Lewis antigens in the decidua and association with inappropriate implantation in uRM remains unknown. To clarify this issue, our study aimed to assess whether these Lewis antigens and pertinent glycosyltransferases are altered in the decidua of uRM patients, and elucidate their potential link with the impaired selection ability of ESCs in uRM.

## Methods

### Patient

Placental samples of RM (n = 15) and legally terminated normal pregnancies (NC, n = 10) were chosen from the tissue bank of the Department of Obstetrics and Gynecology of LMU Munich as previously described (21). Details of the study population are summarized in **Table 1**.

**Table 1:** Demographic and clinical characteristics of the study population.

Characteristics	Normal Control (n=10)	Recurrent Miscarriage (n=15)	*P* value
Maternal age (years)^a^	31.20 ± 2.86	33.50 ± 3.45	0.09
Gestation age (weeks)^a^	9.63 ± 2.40	9.51 ± 1.24	0.87
Gravidity^b^	2 (1-3)	3 (2-6)	0.02
Parity^b^	2 (0-4)	0 (0-2)	0.25

^a^Data represent mean ± S.D. ^b^Data presented as median (range).

### Immunohistochemistry

Paraffin-embedded placental tissue slides were dewaxed in xylol and rehydrated through descending ethanol. Endogenous peroxidase activity was inhibited in 3% H_2_O_2_ for 20 min followed by antigen retrieval in a pressure pot with 0.1mM sodium citrate and 0.1mM citric acid buffer. The slides were blocked with Reagent 1 (ZytoChem Plus HRP Polymer System (mouse/rabbit), Zytomed Berlin, Germany) for 5 min before application of the primary antibodies (details are shown in **Table 2**) for 16 h at 4°C. PBS (pH = 7.4) washing was performed between each step. Subsequent incubation with post block (Reagent 2) for 20 min and with HRP polymer (Reagent 3) for 30 min was applied following the manufacturer’s instructions. The specific antibody binding was visualized with 3,3’-diamionbenzidine substrate-chromogen system (Dako, Denmark) and counterstained with Hemalaun. Certain species-specific isotype control antibodies (Dako) were used for negative control staining. The staining results were analyzed using semi-quantitative immunoreactive score (IRS) as previously described (22).

**Table 2:** Details of primary antibodies used in this study.

Antibody	Isotype	Clone	Source	Dilution	Application
E-Selectin	Mouse IgG	Monoclonal	Calbiochem	1:50	IF
L-Selectin	Goat IgG	Polyclonal	R&D Systems	1:20	IF
P-Selectin	Rabbit IgG	Polyclonal	Sigma-Aldrich	1:50	IF
sLeA	Mouse IgG	Monoclonal	Calbiochem	1:80/50/50/50	IHC/IF/FCM/ICC
sLeX	Mouse IgM	Monoclonal	BD Pharmingen	1:200	IHC
LeX	Mouse IgM	Monoclonal	Novocastra	1:200	IHC
			Calbiochem	1:100/100	FCM/ICC
LeY	Mouse IgM	Monoclonal	LSBio	1:50	IHC
EpCAM	Goat IgG	Polyclonal	R&D Systems	1:100	IF
FUT1	Rabbit IgG	Polyclonal	Invitrogen	1:400	IHC
FUT3	Rabbit IgG	Polyclonal	Abcam	1:200	IHC
FUT4	Rabbit IgG	Polyclonal	Prosci	1:100	IHC
ST3GAL3	Rabbit IgG	Polyclonal	Invitrogen	1:500	IHC
ST3GAL4	Rabbit IgG	Polyclonal	Sino Biological	1:100	IHC
ST3GAL6	Rabbit IgG	Polyclonal	Novus Biologicals	1:100	IHC
NEU1	Rabbit*	Polyclonal	Sigma-Aldrich	1:50	IHC

*Isotype is not mentioned in the datasheet. IF, immunofluorescence; IHC, immunohistochemistry; FCM, flow cytometry; ICC, immunocytochemistry; EpCAM, Epithelial cell adhesion molecule.

### Immunofluorescence

Placenta tissue slide was processed in the same experimental steps as for immunohistochemistry until the step of blocking: Ultra V Block solution (Lab Vision, USA) was applied for 5 min and then incubated with specific primary antibodies for 16 h at 4°C. After washing twice in PBS, slides were incubated with certain fluorescent secondary antibodies (**Table 3**) according to the host species of different primary antibodies for 30 min at room temperature in darkness. Finally, slides were embedded in Vectashield^®^ mounting medium with DAPI (Vector Laboratories, USA) for nucleus staining after washing and drying. Cell smears were prepared on slides in quadriPERM culture dish (SARSTEDT, Germany). Cells on the slides were fixed with ethanol/methanol solution for 15 min followed by 5 min incubation with Ultra V Block solution, subsequent steps are the same as tissue slide. Double immunofluorescent staining of placenta tissue was examined using Leica SP8 confocal system (Leica Microsystems, Germany) and processed with LAS X software (Leica Microsystems, Germany). Immunofluorescent staining of cell smears was examined by Axioskop photomicroscope (Carl Zeiss Microscopy GmbH, Germany) and processed with ZEN Blue software (Carl Zeiss Microscopy GmbH, Germany).

**Table 3:** Details of fluorescent secondary antibodies used in this study.

Antibody	Source	Dilution	For primary antibody
Goat anti-mouse, Alexa Fluor 488	Dianova	1:100	E-Selectin
Donkey anti-goat, Alexa Fluor Plus 488	Invitrogen	1:300	L-Selectin
Donkey anti-rabbit, DyLight 488	Invitrogen	1:100	P-Selectin
Donkey anti-mouse, Alexa Fluor 488	Invitrogen	1:200	sLeA
Donkey anti-goat, Cy2	Dianova	1:100	EpCAM

### Cell Lines and Culture

Endometrial cell line RL95-2, general model for receptive epithelial cells (23), and trophoblast cell line HTR-8/SVneo, derived from first trimester villous trophoblast with characteristics of villous cytotrophoblast and extravillous trophoblast (23), were obtained from American Type Culture Collection (ATCC), and were maintained in RPMI 1640 medium + GlutaMAX™ (Gibco, USA) supplemented with 10% fetal bovine serum (Gibco, USA) in a 5% CO_2_ incubator at 37°C. Medium was refreshed every second day.

### Cell Transfection

RL95-2 cells were cultured in 6-well plates (10^6^ cells per well, Corning Inc., USA) until 70% confluency and transiently transfected with Silencer Select Pre-designed FUT3 siRNA (12 pmol per well, Thermo Fisher Scientific, USA) with Lipofectamine RNAiMAX (Thermo Fisher Scientific, USA) in Opti-MEM (Gibco, USA) according to the manufacturer’s instructions. In the meantime, AllStars Negative Control siRNA (Qiagen, Germany) transfected cells were used as the control. Cells were harvested after 48 h culture for RT-qPCR and 72 h culture for flow cytometry detection, respectively. For adhesion assay, after 24 h siRNA transfection, cells were replated into 8-well chamber slides (Millipore, Germany) at 2×10^5^ per well and cultured for another 48 h until spheroids adhesion.

### RNA Extraction, Reverse Transcription, and RT-qPCR

Total cellular RNA was extracted with NucleoSpin^®^ RNA Mini kit (MACHEREY-NAGEL, Germany) according to manufacturer’s instructions. First strand cDNA was prepared from the total RNA (1 µg) using Oligo dT primers and Reverse Transcriptase (Biozym, Germany). For RT-qPCR, cDNA samples were mixed with FAM-labeled TaqMan probes (β-actin: Hs99999903_m1; FUT3: Hs01868572_s1) from Thermo Fisher Scientific (USA) and TaqMan Fast Universal PCR Master Mix (Applied Biosystems, USA), followed by amplification with 7500 Fast Real-Time PCR system (Applied Biosystems, USA) according to manufacturer’s protocol. Results were calculated by the comparative CT method, with relative transcript levels determined as 2^-ΔΔCT^.

### Flow Cytometry Analysis

Detection of cell surface Lewis epitopes was performed by indirect fluorescence. Briefly, RL95-2 cells were detached from the 6-well plate with the Accutase solution (STEMCELL Technologies, USA) digestion, 10^6^ cells were stained with respective primary antibodies (**Table 2**) diluted in DPBS-0.5% Bovine Serum Albumin (BSA). After washing twice, cells were incubated with 1:300 diluted fluorochrome-conjugated secondary antibody Alexa Fluor 488 goat anti-mouse IgG (Dianova, Germany). Three independent assays for each sample were analyzed with a BD Accuri™ C6 Cytometer (BD Biosciences, USA).

### Pro-Implantation Cytokine IL-1β Treatment

To form a complete endometrial monolayer, RL95-2 cells were firstly cultured in T25 flask with or without 20 ng/ml recombinant human IL-1β (R&D Systems, USA) for 48h to yield enough number of cells. These treated and untreated cells were then subcultured into 6-well plates at a density of 10^6^ per well for flow cytometry detection after 48 h, or into 8-well chamber slides for immunocytochemistry (10^5^ per well) and *in vitro* implantation assay (2×10^5^ per well) after 48h with or without IL-1β treatment. Thus, RL95-2 cells were continuously treated with IL-1β for 4 days in total.

### Immunocytochemistry (ICC)

The IL-1β treated RL95-2 cells for immunostaining were prepared in 8-well chamber slides as above explained. Cells on the slides were fixed with ethanol/methanol solution for 15 min. Following steps using ZytoChem Plus HRP Polymer System reagents were the same as immunohistochemical staining except in the last step, slides were not rehydrated in ascending ethanol gradients and were covered with Aquatex^®^ mounting medium (Merck, Germany).

### *In Vitro* Implantation Assay

The RL95-2 cell monolayers were prepared in 8-well chamber slides as above explained. HTR-8/SVneo cells spheroids were created with modified hanging drops method (24). Briefly, 2×10^4^ cells per 30 µL drop supplemented with regular culture medium were plated onto the lid of Petri dishes (40 drops/Petri lid). The lid was inverted over the bottom of Petri dish filled with 5ml DPBS. Hanging drops were cultured for 20 h until the spheroids were properly formed, not over aggregated. At the end of spheroids preparation, they were gently collected from the Petri lid to a 50ml Falcon tube and rinsed with complete culture medium. Spheroids suspension was then passed through 200 µm and 100 µm sieve size cell strainer (pluriSelect, Germany) to select spheroids of size between 100 and 200 µm which is similar to human implantation blastocysts.

Selected spheroids were gently transferred onto each well of confluent RL95-2 cells in 8-well chamber slides, and co-cultured for 2 h for adhesion. For the blockade experiment, RL95-2 monolayer cells were pretreated with different concentrations of anti-sLeA/LeX antibody or negative control IgG/M antibody (Dako, Denmark) for 1 h before co-culture. A total number of transferred spheroids in each well were firstly counted, then nonadherent spheroids were aspirated off, washed with DPBS twice and the number of attached spheroids was counted under microscope (Leica Microsystems, Germany). The results were presented as percentage of adhesion spheroids. All experiments were performed in three replicates on three separate days.

### Statistical Analysis

Graphpad Prism 8 (Graphpad Software Inc., USA) was used to analyze statistical differences between groups. The normality of data was tested with Shapiro-Wilk’s test. Two-tailed Student’s *t*-test (for normally distributed data) or Mann-Whitney U test (for non-normally distributed data) was performed for two groups comparison. *P <*0.05 was determined to be significant.

## Results

### Upregulated Expression of Lewis Antigens and Associated Key Modulators in uRM

**Figure 1:**
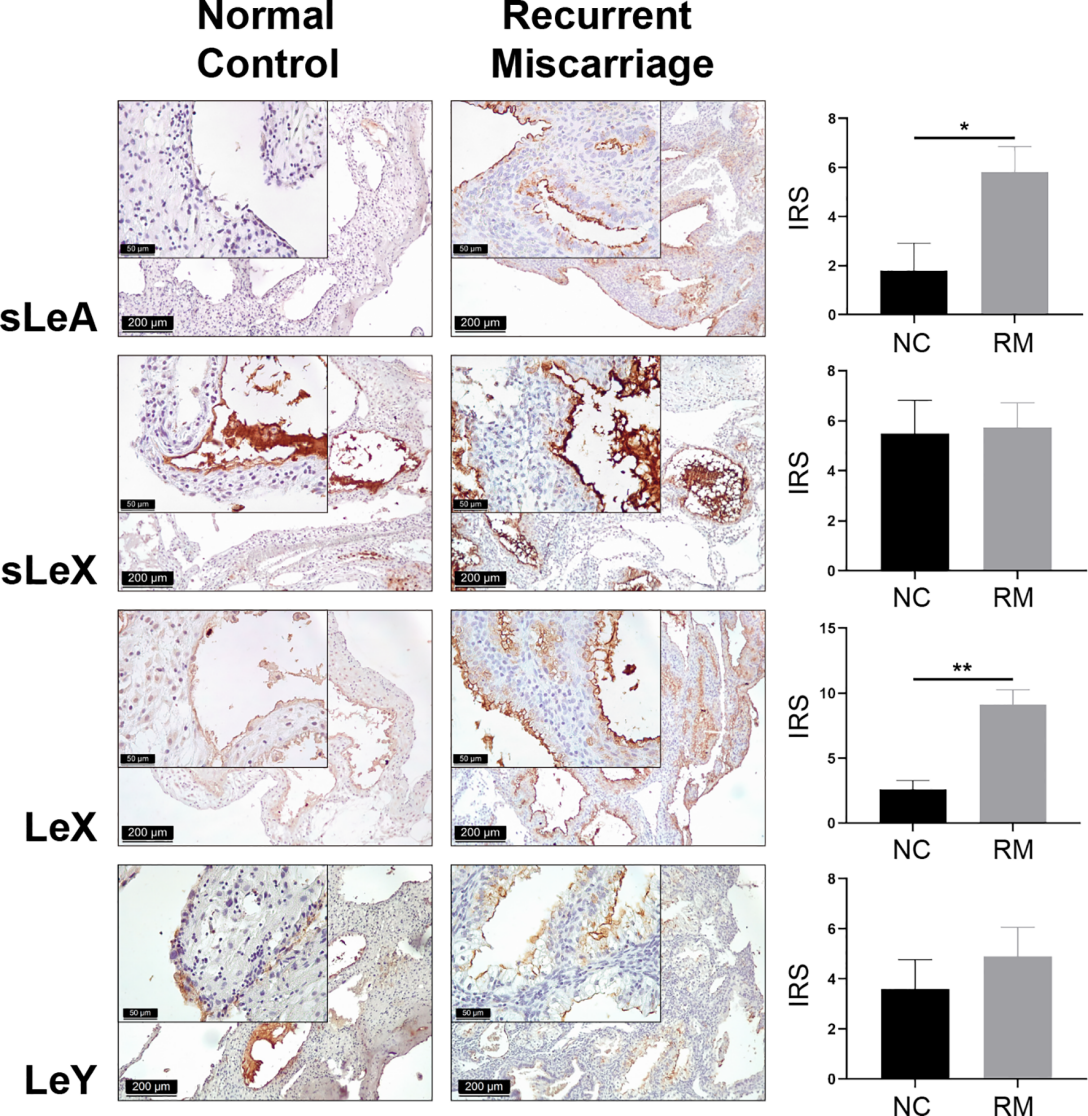
Immunohistochemical staining of Lewis antigens in the decidua of Normal Control group (NC = 10) and Recurrent Miscarriage group (RM = 15) of placenta tissues, demonstrating higher expression of sLeA and LeX in RM than NC. IRS, immunoreactive score. Error bars, S.E.M. *P < 0.05, **P < 0.01.

**Figure 2:**
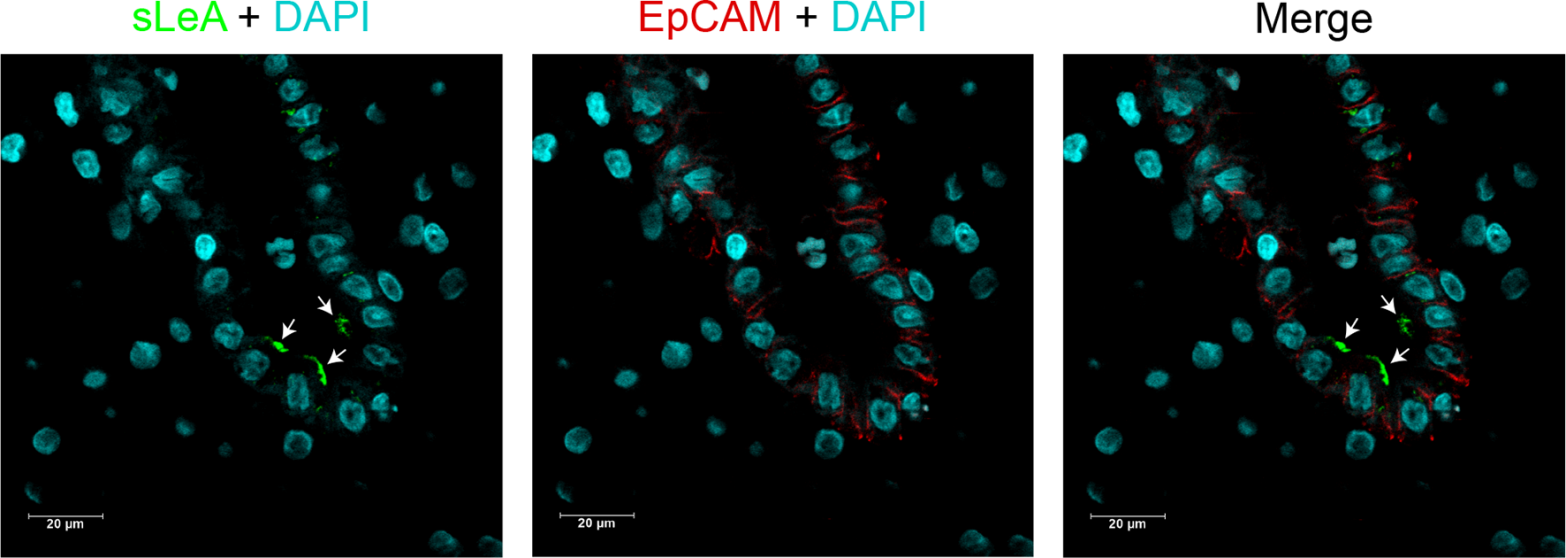
Cellular localization of sLeA in epithelial cells of placenta tissue. sLeA (green) and epithelial cell marker EpCAM (red) were double stained, the confocal images show that sLeA localizes at the apical membrane of epithelial cells as white arrows indicate.

Lewis antigens expression localized mainly in the decidual luminal and glandular epithelium, sparsely in the stromal cells (**Figure 1**). To display the cellular localization of Lewis antigens in the epithelial cells more precisely, double immunofluorescent staining of sLeA and epithelial marker EpCAM (Epithelial cell adhesion molecule) was performed. As the confocal images indicate, sLeA localizes at the apical membrane of epithelial cells (**Figure 2**). The expression of sLeA and LeX both were prominently higher in the RM group than the NC group (*P <*0.05 and *P <*0.01, respectively). While sLeX and LeY showed no significant differences between groups.

**Figure 3:**
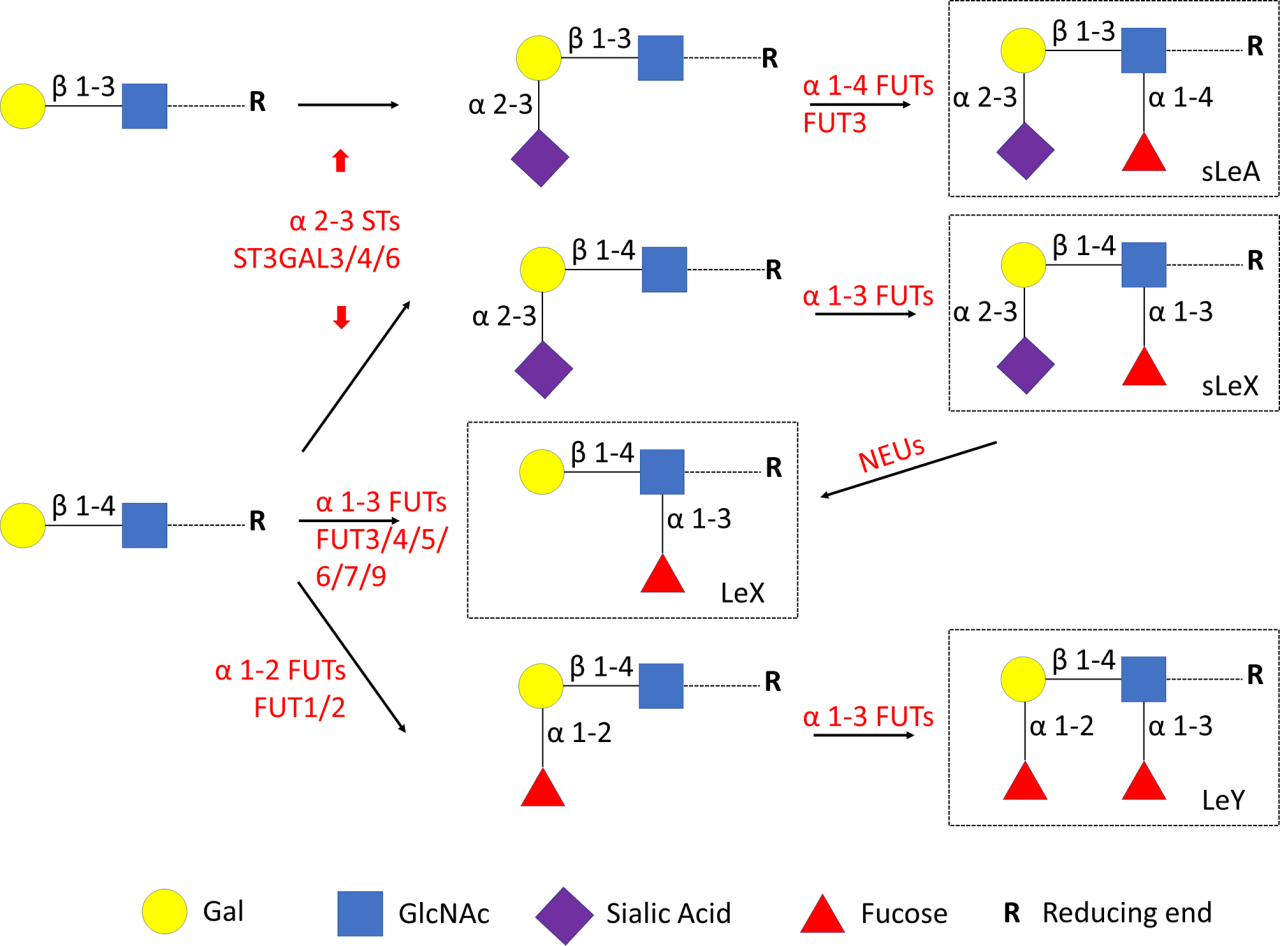
Lewis antigens biosynthesis. FUTs, fucosyltransferases. STs, sialyltransferases. NEUs, neuraminidases. Gal, galactose. GlcNAc, N-acetylglucosamine.

**Figure 4:**
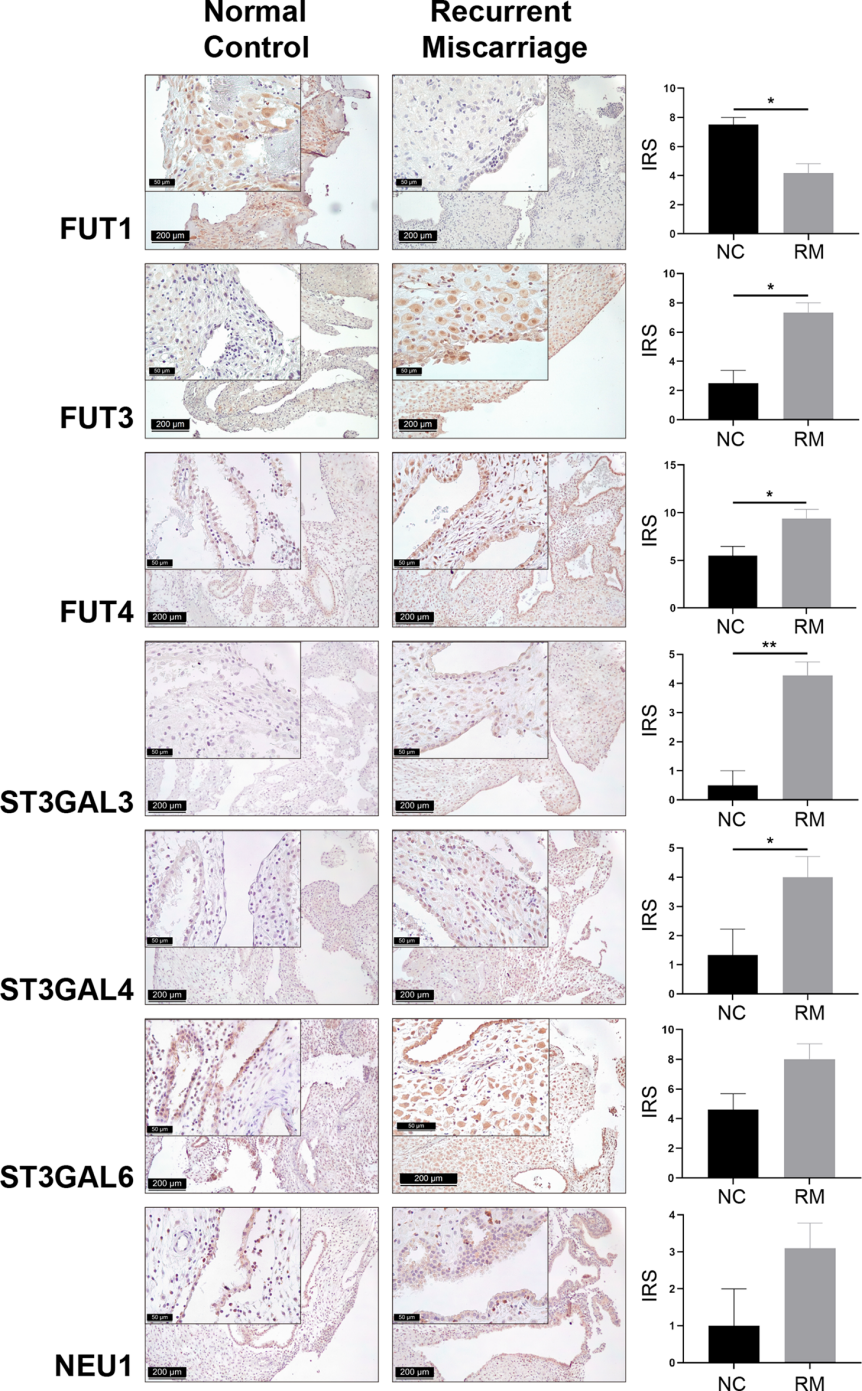
Immunohistochemical staining of seven Lewis antigens associated key modulators in the decidua of Normal Control group (NC = 10) and Recurrent Miscarriage group (RM = 15) of placenta tissues, demonstrating higher expression of FUT3/4 and ST3GAL3/4 and lower expression of FUT1 in RM than NC. IRS, immunoreactive score. Error bars, S.E.M. *P < 0.05, **P < 0.01.

Cellular glycan maintenance is a complex process, in which FUTs and STs are responsible for the synthesis of fucose residues and sialic acid, while NEUs catalyze the removal of sialic acid moieties (**Figure 3**). To identify the molecular mechanism underlying dysregulated Lewis antigens in uRM, we also evaluated the expression of pertinent glycosyltransferases FUT1/3/4, ST3GAL3/4/6, and NEU1. Here, we found these glycosyltransferases were expressed in both stromal and epithelial cells, and generally higher in the RM group except FUT1 (**Figure 4**). FUT3 and FUT4 were significantly higher in the RM group than the NC group (both *P <*0.05). Similarly, ST3GAL3 and ST3GAL4 in the RM group were prominently higher than the NC group (*P <*0.01 and *P <*0.05, respectively). Notably, FUT1 expression was significantly higher in the NC group than the RM group (*P <*0.05). ST3GAL6 and NEU1 were slightly higher in the RM group, but the differences were not prominent (*P* = 0.083 and *P* = 0.084, respectively). Negative controls staining is shown in **Supplementary Figure 1**.

### Spheroids Formation, Adhesion and Selectins Expression in HTR-8/SVneo Cells

**Figure 5:**
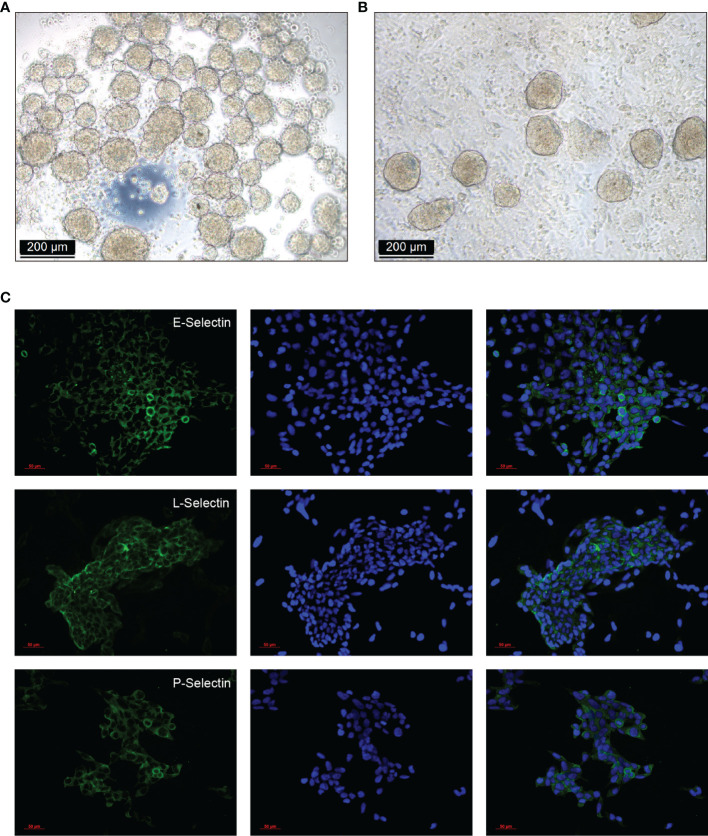
Spheroids formation, adhesion, and selectins expression in HTR-8/SVneo cells. **(A)** HTR-8/SVneo cells spheroids were created with the modified hanging drops method, 2×10^4^ cells per 30 µL drop supplemented with regular culture medium were plated onto the lid of Petri dishes (40 drops/Petri lid). Plenty of spheroids were formed after 20 h culture. **(B)** Cell strainer selected spheroids (100 - 200 µm) were gently transferred onto each well of confluent RL95-2 cells in 8-well chamber slides, and co-cultured for 2 h for adhesion. **(C)** Expression of Lewis antigens putative ligands E, L, and P selectins in HTR-8/SVneo cells detected by immunofluorescent staining.

Plenty of spheroids were formed after 20 h culture with modified hanging drops method (**Figure 5A**). After co-cultured for 2 h for adhesion, cell strainer selected spheroids (100 - 200 µm) attached to the confluent RL95-2 cells (**Figure 5B**). Lewis antigens putative ligands E, L, and P selectins could be detected in HTR-8/SVneo cells by immunofluorescent staining (**Figure 5C**).

### FUT3 Knockdown Decreased sLeA Expression and Spheroids Attachment in RL95-2 Cells

**Figure 6:**
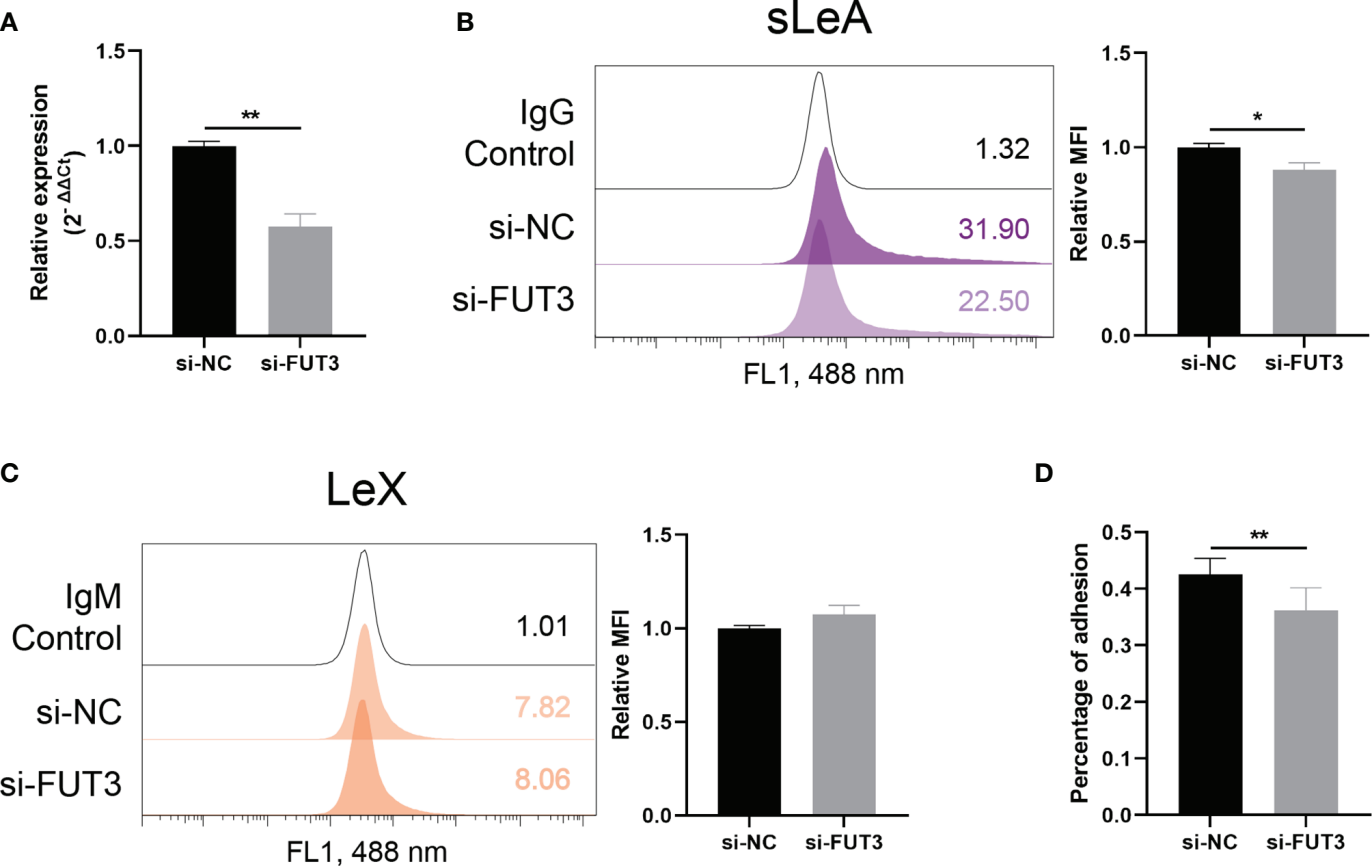
FUT3 knockdown in RL95-2 cells. **(A)** RT-qPCR demonstrating efficiency of siRNA knockdown of FUT3 in RL95-2 cells. **(B, C)** Flow cytometry demonstrating reduced expression of sLeA but unchanged expression of LeX after the knockdown of FUT3 in RL95-2 cells, relative mean fluorescence intensity (MFI) is used for statistical analysis, numbers inside the frame denote the positive cells (%) in a representative experiment. **(D)** *In vitro* implantation assay suggesting suppressed trophoblast spheroids adhesion to endometrial monolayer after the knockdown of FUT3 in RL95-2 cells. The total number of transferred spheroids in each well was firstly counted, then nonadherent spheroids were aspirated off, washed with DPBS twice and the number of attached spheroids was counted again under microscope. The results were presented as percentage of adhesion spheroids. Statistical analysis was performed in three independent experiments by *t* test. Error bars, S.E.M. si-NC, negative control siRNA. *P < 0.05, **P < 0.01.

Modulatory effect of FUT3 on the expression of Lewis antigens in RL95-2 cells was confirmed by siRNA knockdown experiments (**Figure 6**). Expression of cell surface sLeA was decreased after FUT3 knockdown in RL95-2 cells (*P <*0.05, **Figure 6B**), while the expression level of LeX remained unchanged (**Figure 6C**). The percentage of attachment of HTR-8/SVneo spheroids to endometrial monolayers was also significantly suppressed by FUT3 knockdown (*P <*0.01, **Figure 6D**).

### Anti-sLeA Suppressed IL-1β Induced Trophoblast Spheroid Attachment to Endometrial Cells

**Figure 7:**
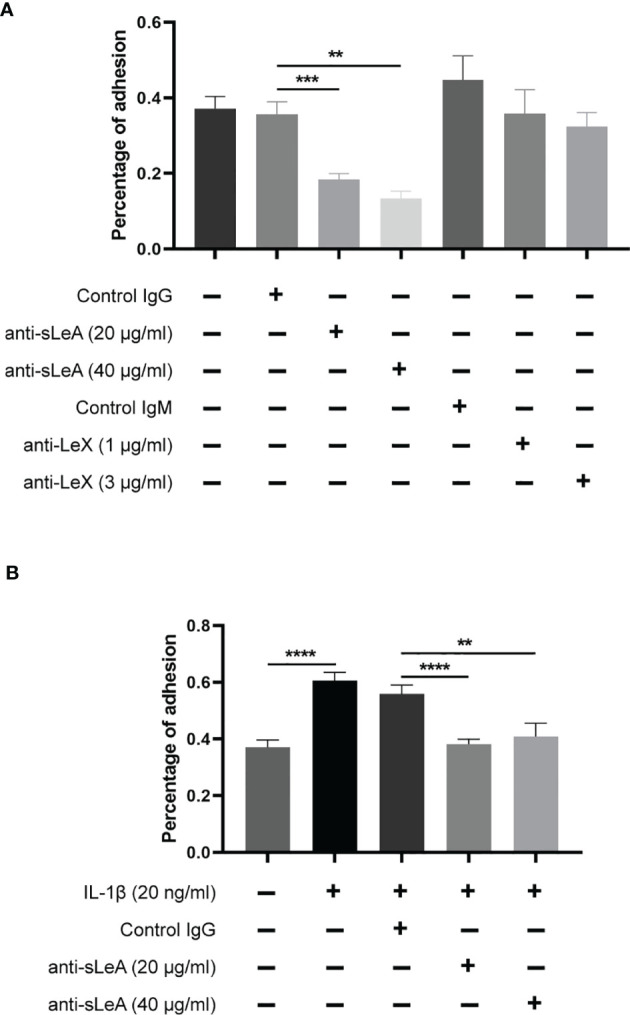
Antibody blockade and IL-1β stimulated *in vitro* implantation assay. **(A)** RL95-2 monolayer cells were pretreated with different concentrations of anti-sLeA/LeX antibody or negative control IgG/M antibody for 1 h before co-culture. The total number of transferred spheroids in each well was firstly counted, then nonadherent spheroids were aspirated off, washed with DPBS twice and the number of attached spheroids was counted again under microscope. The results were presented as percentage of adhesion spheroids. Anti-sLeA antibody significantly suppressed the spheroids attachment, while anti-LeX antibody only slightly suppressed the attachment but the difference was not prominent. **(B)** RL95-2 cells were continuously cultured at the presence of IL-1β (20ng/ml) for 4 d before the *in vitro* implantation and blockade assay. IL-1β treatment markedly increased the trophoblast spheroids attachment, and the induced attachment could be dramatically suppressed by anti-sLeA antibody. Statistical analysis was performed in three independent experiments by *t* test. Error bars, S.E.M. **P < 0.01, ***P < 0.001, ****P < 0.0001.

To clarify the involvement of sLeA and LeX in the process of blastocyst adhesion, specific antibody blockade was applied in the *in vitro* implantation assay. Blocking sLeA with anti-sLeA antibody significantly suppressed the attachment of spheroids to endometrial monolayers, while the blockade efficacy showed no differences between 20 and 40 µg/ml antibody concentration (**Figure 7A**). Anti-LeX antibody slightly suppressed spheroid attachment but the difference was found to be not significant (**Figure 7A**). Here, we also found that IL-1β treatment markedly increased the trophoblast spheroids attachment to endometrial cells monolayers (*P <*0.0001, **Figure 7B**), confirming its function as an implantation promoting factor in the *in vitro* model. Interestingly, blocking sLeA could still suppress the IL-1β induced attachment and the blockade efficacy also did not differ between 20 and 40 µg/ml antibody concentration.

### Induction of LeX Expression in Endometrial Cells by IL-1β

**Figure 8:**
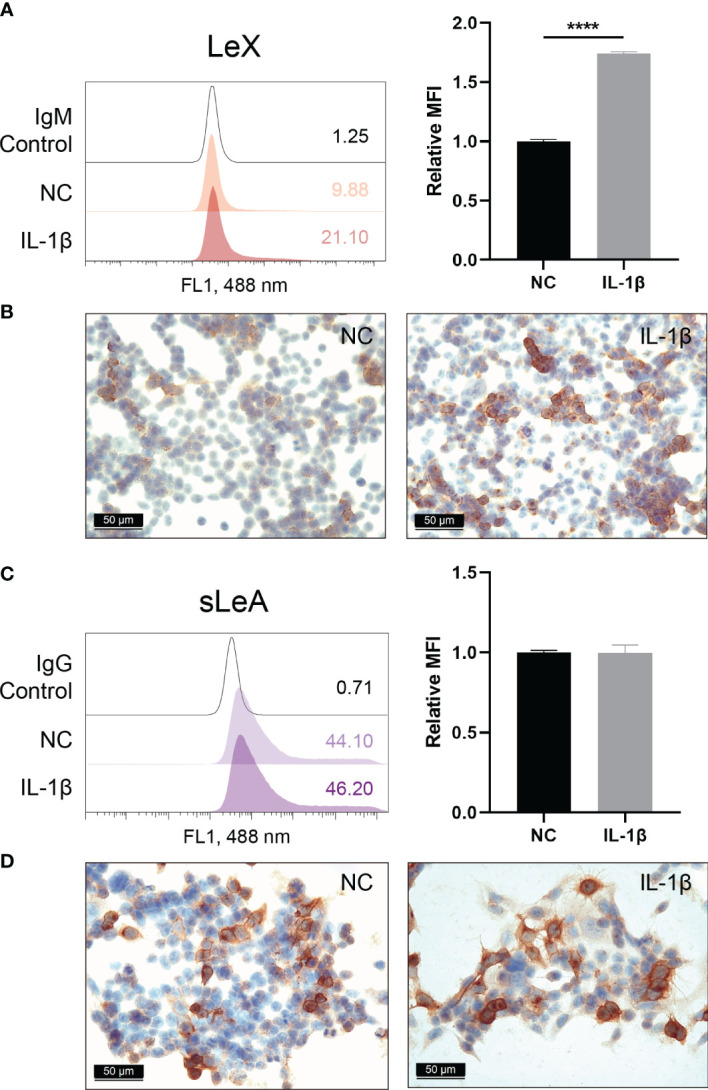
Cell surface expression of LeX and sLeA after IL-1β treatment in RL95-2 cells. Flow cytometry detection of LeX **(A)** and sLeA **(C)**, relative mean fluorescence intensity (MFI) is used for statistical analysis, numbers inside the frame denote the positive cells (%) in a representative experiment. Immunocytochemical staining of LeX **(B)** and sLeA **(D)** in RL95-2 cells without and with IL-1β treatment (20ng/ml). Statistical analysis was performed in three independent experiments by *t* test. Error bars, S.E.M. NC, untreated. ****P < 0.0001.

Influence of IL-1β treatment on the expression of sLeA and LeX in endometrial cells was investigated by flow cytometry. We found significantly enhanced expression of LeX following stimulation with IL-1β (*P <*0.0001), with a 1.74-fold increase in mean fluorescence intensity (MFI) values compared to untreated cells (**Figure 8A**). ICC staining also confirmed the induction of LeX expression in RL95-2 cells treated with IL-1β (**Figure 8B**) while changes in the expression level of cell surface sLeA were not detected (**Figures 8C, D**).

## Discussion

Pregnancy is characterized by the intimate interactions between a competent embryo and a receptive endometrium. Appropriate blastocyst implantation is widely considered as a crucial factor for achieving successful pregnancy.

Monthly fecundity rates (MFRs) refer to the time taken to achieve pregnancy, which is used to measure the probability of conceiving within one menstrual cycle (25). The average MFR in humans is relatively low at around 20%, while studies have shown that 32-40% of women experiencing RM have exceptionally high pregnancy rates with MFR > 60% (8). However, increased pregnancy rates do not result in increased live birth rates but repeated miscarriages, this paradox leads to the hypothesis of impaired natural embryo selection in RM (4). Biopsies analysis of mid-secretory endometrium from RM revealed decreased expression of mucin 1, an anti-adhesion molecule that maintains the barrier function of endometrial surface (26). *In vitro* decidualization of ESCs from RM manifested by attenuated production of prolactin and enhanced expression of prokinectin-1, a cytokine that facilitates implantation (27). Moreover, ESCs from RM cannot distinguish trophoblast signals between high- and low-quality embryos, resulting in increased migration behavior than normal fertile women (10). Thus, impaired endometrial barrier, aberrant decidualization associated prolonged “window” of receptivity, and less selective decidual phenotype in RM together allow the implantation of low viable embryos which are destined to fail.

Glycoconjugates at the fetomaternal interface play a vital role in mediating the adhesion of blastocyst to the endometrium. SLeA and sLeX are common selectin oligosaccharide ligands, their expression in the endometrium reaches the highest level during the window of implantation (12). Similarly, LeY was highest expressed in the mid-secretory phase during menstrual cycle, while expression of LeX peaked at early- to mid-proliferative phases (28). Biosynthesis of Lewis antigens requires the sequential addition of sialic acid and fucose by α 2-3 STs and FUTs, respectively (22). Genbacev et al. firstly clarified the role of L-selectin/sLeX adhesion system in trophoblast implantation (18). Later functional studies showed that both ST3GAL3 and FUT7 transfection could facilitate the embryo adhesion through upregulating sLeX (19, 29, 30). Integrin αvβ3, biomarker of endometrial receptivity, was reported to be the carrier of LeY, and knocking down FUT4 significantly decreased the adhesion of trophoblast JAR cells to RL95-2 cells by reducing the expression of LeY (20).

Despite the fact that sLeX and LeY play important roles in blastocysts adhesion, our study found no alterations of their expression in the decidua of RM patients, but sLeA and LeX showed dramatic upregulation. SLeA has been intensively studied in various cancers, it promotes the metastasis and malignant transformation by interacting with selectins (14). Similar to sLeX, highest level of sLeA in the endometrium through menstrual cycle is also noticed during the implantation stage, while its significance in the process of human implantation is not fully understood. Our *in vitro* implantation assay showed that anti-sLeA can prominently inhibit the adhesion of trophoblast spheroids to endometrial monolayers, indicating that sLeA also mediates the blastocyst implantation process. Thus, aberrantly elevated sLeA may be a novel marker for the hyper-receptive/less selective endometrium in RM. LeX is a carbohydrate cell adhesion molecule, it plays a crucial role in human embryogenesis and neutrophil transepithelial migration (31, 32). Upregulated LeX usually leads to metastasis and decreased survival in a variety of cancers (33–35). While the absence of LeX on human glioma cells is thought to be the reason that extraneural metastasis of brain tumors is rare (36). LeX expression was also identified in the normal endometrium and can be upregulated by progesterone (37). To test whether LeX is involved in trophoblast adhesion, anti-LeX antibody was used in the *in vitro* implantation assay, while no significant decrease was shown in the spheroids adhesion. This might explain why the highest expression of LeX in the endometrium does not occur at the implantation stage during menstrual cycle. Meanwhile, we also noticed that the basal expression of LeX in RL95-2 cells is around 10% (LeX positive cells) as flow cytometry detected (**Figure 8A**), thus model with RL95-2 cells may not adequately represent LeX expression usually found in receptive endometrium thereby confounding the results of the spheroids adhesion assay. The potential significance of LeX in blastocyst implantation should not be completely excluded, further investigations are merited.

The expression of potential glycosyltransferases FUTs, α2-3 STs, and NEUs were also validated in the decidua of RM. FUT3/4 and ST3GAL3/4 were significantly upregulated in the RM group, while FUT1 was downregulated. ST3GAL6 and NEU1 did not prominently differ between groups. FUT3 is the only fucosyltransferase that generates sLeA through an α1-4 linkage addition of fucose moieties (38). Hepatitis B virus X protein targets FUT3 for the production of sLeA (39). FUT3 was identified as the key enzyme for sLeA synthesis in human intestinal epithelial cells (IECs), its transfection of sLeA-deficient IECs resulted in robust expression of sLeA (40). Here we also found significantly upregulated FUT3 in the decidua of RM and FUT3 knockdown in RL95-2 cells decreased the cell surface expression of sLeA and suppressed the spheroids adhesion, which indicate that FUT3 may account for the aberrantly elevated sLeA and endometrial hyper-receptivity in RM. FUT3 also exhibits α1-3 fucosyltransferase activity that contributes to sLeX expression (11, 41). While upregulated FUT3 in the decidua of RM does not result in higher expression of sLeX as displayed in our study, this might be explained by different catalytic preferences of α1-3 FUTs in different diseases (14). Other α1-3 FUTs like FUT5/6/7/9, which also contribute to the synthesis of sLeX, were not extensively investigated in this study but might be the main modulators for sLeX at the fetomaternal interface. As a member of the human neuraminidases family, NEU1 catalyzes the removal of sialic acid moieties from glycoproteins and glycolipids, it dramatically enhances LeX production *via* sLeX desialylation during human myeloid differentiation (17). Expression of NEU1 in the RM group was mildly elevated but did not significantly differ from the NC group (*P* = 0.084), which is probably due to the relatively small sample sizes. Whether NEU1 is really upregulated in RM and collaborates with FUT3 results in balanced sLeX and enhanced LeX expression needs to be revealed in larger studies. Besides, α1-3 FUTs including FUT3 and FUT4 can directly catalyze the synthesis of LeX by adding fucose to the N-acetylglucosamine (GlcNAc) residue of glycans through an α1-3 linkage (38, 42). FUT4 expression in the endometrium is dynamically changing during menstrual cycle and reaches the highest level during implantation stage, progesterone shows dramatical induction of FUT4 expression (43). While FUT3 knockdown did not affect the expression level of LeX in RL95-2 cells, other α1-3 FUTs, like elevated FUT4 may explain the higher expression of LeX and contribute to the hyper-receptive endometrium in RM. In the synthesis of LeY, FUT1 mediates the first fucose addition to galactose (Gal) followed by second fucose addition to GlcNAc catalyzed by FUT3/4 (**Figure 3**), upregulated FUT3/4 and downregulated FUT1 in the decidua may result in an unchanged level of LeY in RM.

ST3GAL3/4/6 belongs to the α2-3 STs family that catalyzes the addition of sialic acid to the Gal residue of glycans, yielding sLeA/X (11, 15, 44–46). In humans, ST3GAL3 exhibits a preferential effect on type I disaccharides for the synthesis of sLeA (47). ST3GAL3 expression in the endometrium at secretory phase is significantly higher than the proliferative phase, its downregulation decreases the adhesion of trophoblast cells to endometrial cells (30). ST3GAL4 has been reported to be the major STs regulating sLeX in human myeloid leukocytes (48). This study found prominently higher expression of ST3GAL3/4, but not ST3GAL6, in RM than the control group, which might be also responsible for elevated sLeA expression. Notably, FUTs and STs compete for the same substrate, selective inhibition of fucose addition facilitates greater sialylation (11, 48, 49). Therefore, downregulation of FUT1 may not only maintains the stable level of LeY, but could potentially also allow for more substrates to be modified into LeX and sLeA by FUT3/4 and ST3GAL3/4, respectively.

IL-1β was reported to play pivotal roles in human embryo implantation and has been widely acknowledged as a pro-implantation cytokine (50), it induces endometrial expression of adhesion molecule integrin β3 and promotes the extravillous motility (51, 52). Since anti-sLeA blocked the adhesion of trophoblast to endometrial monolayers at a basal level, we further investigated if anti-sLeA could also exert anti adhesion effect at the presence of IL-1β stimulation. Importantly, while IL-1β treatment of RL95-2 cells, in line with previous studies, significantly enhanced the spheroids adhesion rate, anti-sLeA did partially block the IL-1β induced adhesion. Pro-inflammatory cytokines induced glycosylation alterations have been reported in many studies, IL-8 induces sLeA expression in human pancreatic cancer cells (53), TNF-α stimulation prominently increases sLeA reactivity in gastric adenocarcinoma cells while IL-1β results in mild enhancement of sLeA and LeX but does not reach significance (54). In this study, IL-1β treatment of RL95-2 cells showed no alterations in sLeA while significant increase in LeX expression. Hence, sLeA expression is independent of IL-1β signaling but anti-sLeA still exerts sufficient blockade effect on IL-1β induced trophoblast adhesion. This further elucidated the crucial role of sLeA in blastocyst adhesion and anti-sLeA in attenuating the hyper-receptive endometrium in RM. IL-1β induced LeX expression may suggest that previously reported elevated IL-1β in both endometrium and decidua of RM could be a trigger of the upregulated LeX observed in this study (55, 56).

In summary, this study demonstrates a remarkably elevated glycosylation status in the decidua of uRM patients manifested by prominently upregulated sLeA, LeX, FUT3/4, and ST3GAL3/4, which might contribute to the hyper-receptive/less selective endometrium in RM. Involvement of sLeA in the process of blastocyst adhesion is also deciphered with *in vitro* implantation model. Targeting aberrantly elevated sLeA may be applied as a potential strategy to restore the inappropriate implantation in uRM.

## Data Availability Statement

The raw data supporting the conclusions of this article will be made available by the authors, without undue reservation.

## Ethics Statement

The studies involving human participants were reviewed and approved by Ethics Committee of LMU Munich. The patients/participants provided their written informed consent to participate in this study.

## Author Contributions

UJ and VS conceived of the study and participated in its design and coordination. ZM performed the experiments and statistical analysis and wrote the manuscript. HY and MK performed technical assistance in immunohistochemistry and cell culture experiments. MK, MS, SM, and UJ revised the manuscript for important intellectual content. All authors contributed to the article and approved the submitted version.

## Conflict of Interest

The authors declare that the research was conducted in the absence of any commercial or financial relationships that could be construed as a potential conflict of interest.

## Publisher’s Note

All claims expressed in this article are solely those of the authors and do not necessarily represent those of their affiliated organizations, or those of the publisher, the editors and the reviewers. Any product that may be evaluated in this article, or claim that may be made by its manufacturer, is not guaranteed or endorsed by the publisher.
