# Supplementary material for: Targeting Aberrantly Elevated Sialyl Lewis A as a Potential Therapy for Impaired Endometrial Selection Ability in Unexplained Recurrent Miscarriage
Source: Front Immunol. 2022 Jun 28;13:919193. doi: 10.3389/fimmu.2022.919193 (PMC9273867; doi:10.3389/fimmu.2022.919193)
Supplement: Supplementary file 1 [file DataSheet_1.docx]

***Supplementary Material***


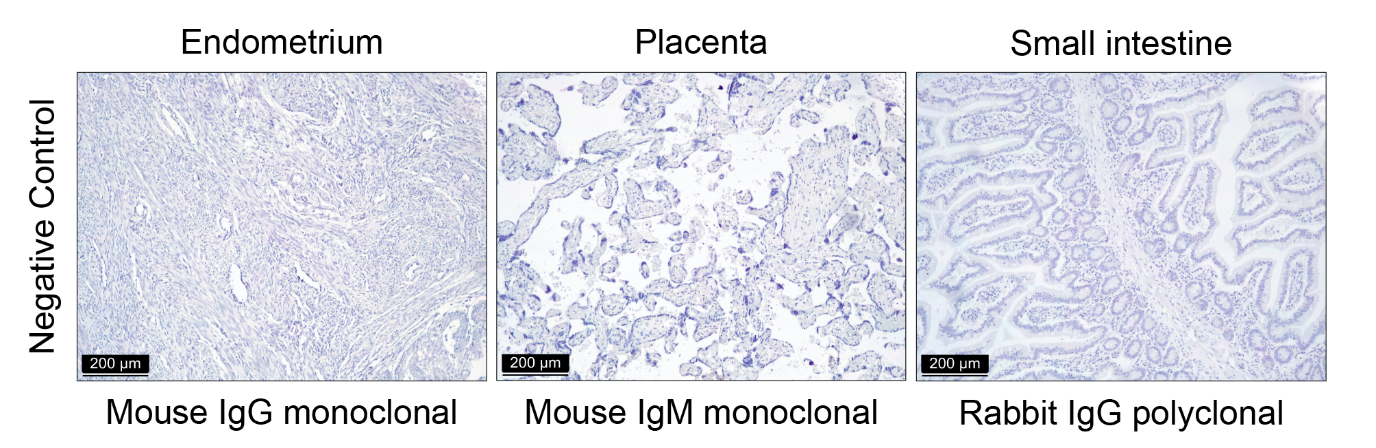


**Supplementary Figure 1.** Immunohistochemical staining of different isotypes of negative controls.
